# DNA Data Marketplace: An Analysis of the Ethical Concerns Regarding the Participation of the Individuals

**DOI:** 10.3389/fgene.2019.01107

**Published:** 2019-11-05

**Authors:** Eman Ahmed, Mahsa Shabani

**Affiliations:** ^1^Center for Biomedical Ethics and Law, Department of Public Health and Primary Care, University of Leuven, Leuven, Belgium; ^2^Clinical Pharmacology Department, Faculty of Medicine, Suez Canal University, Ismailia, Egypt; ^3^Metamedica, Faculty of Law and Criminology, Ghent University, Ghent, Belgium

**Keywords:** genomics, data sharing, incentives, research ethics, privacy

## Abstract

Personal genomic data and the related health data are valuable resources for both public-funded research, and for-profit entities in development of new drugs, therapies, and diagnostic tests. In order to access to large datasets, pharmaceutical and biotech companies have developed partnerships with public and private entities such as direct-to-consumer genetic testing companies to buy genomic and health related databases collected from research participants and customers. Although individuals mainly support data sharing for research purposes, the for-profit nature of such data sharing raises some questions regarding the rights of the data subjects and fairness in sharing benefits. In response, a new generation of sequencing and data sharing startups such as Nebula Genomics, LunaDNA, and EncrypGen are emerging which aim for leaving the data control in the hands of each individual customer. In particular, such so-called “DNA data marketplaces” allow individuals to receive various types of monetary incentives to sequence their genome and share it with interested commercial parties. This paper aims to provide an exploratory and critical review of the ethical challenges related to establishing such marketplaces for genomic and health data sharing. In the view of the growing number of startups developing such marketplaces, a thorough analysis of the relevant ethical concerns is timely and needed.

## Introduction

Personal genomic data and the related health data are valuable resources for both public-funded research, and for-profit entities in development of new drugs, therapies, and diagnostic tests. In order to access to large datasets, pharmaceutical, and biotech companies have developed partnerships with public and private entities such as direct-to-consumer (DTC) genetic testing companies to buy genomic and health related databases collected from research participants and customers.

Most of the customers of DTC companies such as 23andMe opt-in to participate in research activities of the service providers and the downstream data sharing by the companies for research purposes (Hirschler, 2018). The existing studies with customers have revealed that the underlying reasons are mainly out of altruistic motivation to participate in research and help advancement of science (Trinidad et al., 2010; Goodman et al., 2017). However, the for-profit nature of sharing customers’ data by DTC companies has been perceived objectionable by some customers (Skloot, 2015). Notably, by giving consent to research, customers should accept that they acquire no rights to research, products, or profits that are made and may link to their DNA (Ducharme, 2018). This is viewed as unfair where a clear asymmetry in sharing benefits and interests is witnessed.

Moreover, the active participation of the individuals in managing sharing and access to their own genomic and health data in the framework of the current data sharing models is not fully supported. The importance of this matter is recently pronounced by the European Data Protection Supervisor in their statement: “In principle, individuals should be able to decide whether and with whom to share their personal information, for what purposes, for how long, and to keep track of them and decide to take them back when so wished” (European Data Protection Supervisor, 2016).

In response, a new generation of startups are emerging which propose, among others, to leave data control in the hands of each individual customer (Rosenbaum, 2018). These so-called “DNA data marketplaces” propose that people can share their data with companies that are interested to have access to their data for various research leading to product development (Harris, 2018) and receive monetary compensation or incentives (Jones, 2018). Although offering direct incentives to individuals to engage them in genomic data sharing may seem beneficial, this has seen as a sensitive issue drawing a lot of attention in the area of research ethics.

In an effort to address the associated concerns with DNA data marketplaces, this paper provides an exploratory and critical review of the associated ethical challenges related to participation of the individuals through analysis of different arguments discussed in academic papers and gray literature.

## DNA Data Marketplace: Three Examples

In order to illustrate our discussion, we reviewed the information provided in the websites of three startups namely Nebula Genomics, LunaDNA, and EncrypGen, which enable individuals to share their genomic data and related health information and receive various monetary incentives. We also consulted the information published in other websites in relation to the visions, policies, and strategies of these startups.

## Nebula Genomics

Nebula Genomics is a startup established by George Church, plans to “upend the usual way genomic information is owned,” claiming that the current system applied by DTC companies is “very paternalistic” (Harris, 2018). Nebula Genomics is aiming for establishing a “Nebula marketplace,” where those consenting to share their genetic information can earn the cryptocurrency called “Nebula tokens” (Buhr, 2018). In Nebula marketplace, individuals are meant to acquire and store their own genomic sequencing directly from Nebula Genomics (in partnership with Veritas) instead of obtaining the service from a personal genomics company. The Nebula’s business model anticipates that companies and research organizations would be willing to pay for the cost of sequencing in exchange to get access to key medical information of the individuals involved. To this end, a blockchain platform is designed to enable customers to choose how and with whom they want their data to be shared, and to be compensated for it (Morris, 2018).

Moreover, Nebula aims for assisting pharmaceutical companies in recruiting research participants with conditions that are interesting for their current studies, by launching an anonymized search for such patients. Once contacted by the companies, the patients can decide if they will grant access to the companies to their genomic and other medical data (Harris, 2018).

## EncrypGen

EncrypGen is a startup aiming to “bring together genomic data sellers and buyers in one platform” (Wilson, 2019) and “looks forward to solving the problem of retaining customers’ DNA data by DTC companies to be resold to research and development companies” (Matthews, 2018). EncrypGen “Gene-Chain” DNA Data Marketplace connects individual DNA data owners with data buyers and providers of other health related services. The Gene-Chain’s aim is to empower users to store and monetize their genetic data by sharing it with third parties looking to obtain genetic data such as research scientists and pharmaceutical companies (Home–EncrypGen | The DNA Data Marketplace–EncrypGen., 2018).

According to the EncrypGen’s website, the individuals are invited to contribute data: “If you have had your DNA tested you may upload your raw DNA data file and create a Gene-Chain profile now. EncrypGen de-identifies the raw DNA data file by stripping it away from name, email, and other sensitive information. DNA data buyers search Gene-Chain profiles suitable for their projects and purchase de-identified genomic data with DNA tokens” (Buy DNA Tokens–EncrypGen., 2018). In addition, EncrypGen has announced the plans for developing partnerships “with testing companies, analytics software developers, and various parties, like employee health benefits services,” in an attempt to drive more users to the platform and monetize data (Levy, 2018).

## LunaDNA

LunaDNA is a community-owned platform that is created by the Public Benefit Corporation, LunaPBC. LunaDNA offers company shares to individuals for contributing their DNA data as well as uploading their medical reports and lifestyle health activities. Those shares entitle members to a share in the profits from medical research and development. Users are supposed to get different portions of shares depending on the data they provide. For example, if a user donates DNA-targeted genes they will receive 10 shares, but if they submit their whole DNA genome, they will receive 300 shares (Lovett, 2018).

LunaDNA platform is powered by blockchain technology and provides aggregated data to researchers with the consent of the involved individuals (Lovett, 2018). In addition, LunaDNA has announced plans for collaboration with pharmaceutical companies in the future.

## Ethical Concerns

Participation of the individuals in for-profit data contribution startups raises a number of ethical concerns for the rights and interests of the individuals and society in general. While some of these concerns are related to the impact of incentives strategies that such startups utilize on consent and participation in research, the other concerns are related to potential privacy concerns that may arise from use of emerging technologies such as Blockchain.

### Consent-Related Concerns

In the context of DNA data marketplace, the impact of monetary incentives on validity of consent should be thoroughly investigated. We will discuss the consent-related issues here under two major concerns of undue influence and withdrawal of consent.

#### Undue Influence

Informed consent must be obtained from participants under circumstances that minimize the possibility of coercion or undue influence. It is important to evaluate whether or under what research circumstances financial incentives might affect a subject’s judgment, and to what degree the payments induce people to participate while having deep objections (Grady, 2005). For instance, according to the official Institutional Review Board guidebook published by the US Office for Human Research Protections, “an offer is troublesome if it is so attractive [that it] may blind prospective subjects to the risks or impair their ability to exercise proper judgment” (U. S. Department of Health and Human Services, 1993).

The question here is under what circumstances offering financial incentives in exchange for individuals personal and health-related data may threat the validity of the consent and compromise the participant’s ability to respond reasonably, resulting in undue induction the participation. In particular, it is crucial to investigate how both patients and healthy participants, with various socio-economic backgrounds respond to the financial incentives in personal data sharing. In traditional research settings, it is expected that the research ethics committees assess the risks of undue influence that may arise from use of monetary and other incentives to recruit research participants. However, in the context of DNA data marketplaces it is not clear if such ethics oversight is present to assess the ethical underpinnings of offering financial incentives in exchange for individuals’ genomic and health-related data.

Other consent-related concerns are based on the nature of genetic data. Given the commonly shared genetic information among relatives, the involvement of the family members in the process of personal genomic data sharing and consent is a matter of discussion. Should all family members approve sharing and selling of common genetic and health information? And should they all benefit from the shares of the same individual account?

Moreover, provision of monetary incentives may have broader impact on biomedical research and data sharing, by undermining altruistic participation in research. One can argue that public-funded research that does not offer monetary incentives can be negatively impacted as a result of recruitment strategies of DNA data marketplaces.

#### Consent Withdrawal

Research participants should be aware that they have the right to freely withdraw their consent at any time during the research (Edwards, 2005), and voluntary terminate their participation in research (Gabriel and Mercado, 2011), without necessarily providing reasons. Notably, offering financial incentives to individuals for sharing their genomic data could be a barrier to consent withdrawal. In particular, the questions arise about whether individuals can withdraw their consent after receiving various types of financial incentive, such as tokens, shares, or free sequencing (Roberts et al., 2017). The procedure of withdrawal could be much more complex when individuals have already allowed access to their data in return for free sequencing of their genome by interested companies.

For instance, the LunaDNA consent policy informs patients that: “Your continued consent to LunaDNA’ s use of your Shared Data is required for your continued ownership of any shares in LunaDNA issued to you in connection with the contribution of that Shared Data. If you elect to purge Shared Data for which you were issued ownership shares in LunaDNA, LunaDNA will redeem (i.e. cancel) those shares, and may also elect to cancel certain other shares that may have been issued to you. [ … ] If you revoke your consent or delete your account, LunaDNA will redeem all shares issued to you.” (LunaDNA, 2018). The other two startups however have not provided any information on this matter on their website. It is highly recommended that these emergent startups establish clear policies regarding consent withdrawal and communicate that to the participants.

### Blockchain-Based Platforms and Privacy Concerns

Sharing personal genomic data raises considerable privacy and security concerns, due to unique nature of genomic data that contains identifiers which makes the complete de-identification of the data hard if not impossible (Wang et al., 2017). In addition, genomic data can reveal a wide range of sensitive health and non-health related data about the individuals and their family members (Genomeweb, 2018). For example, in a study analyzing Y-chromosome haplotypes together with combining data from genealogical registries, researchers were able to predict the surnames of a number of anonymized participants in the dataset (Gitschier, 2009).

As it is reported above, some of the startups aim for implementing blockchain technology as an approach to better protect genomic and health data, while allowing participatory control on access to the data. Blockchain is an emerging technology of a decentralized, digitized database medium and a public ledger of all transactions in the network (Ozercan et al., 2018). The key feature of a blockchain is the distributed database where the database is present in many copies across several computer systems creating a peer-to-peer network indicating that there is no longer a centralized body controlling access to data (Han et al., 2014; Duan et al., 2016). Arguably, blockchain-based platforms can help to solve the governance problems in sharing genomic data by using technical solutions. These platforms promise their customers to provide distributed data stewardship and control together with provision of effective ways for strengthening data access and ownership agreements (Shabani, 2019). In terms of the security of the networks, although blockchain use is expected to improve data encryption (Weintraub, 2018), no technology is infallible and concerns about possible hacking and breaching of the blockchain system have been noticed by the experts and the platform developers (Erickson, 2019).

Nebula Genomics privacy policy includes that they take a number of organizational, technical, and physical measures to protect the personal information they collect, both during transmission and once received. However they note that, “no security safeguards are 100% secure and we cannot guarantee the security of your information“(Privacy Policy, 2018). Moreover, the questions remain about the compatibility of using such technologies with applicable data protection regulations in different jurisdictions. (Price, 2018).

Finally, the possibility of access by third parties such as for law enforcement purposes should be investigated (Weintraub, 2018). The Nebula Genomics privacy policy includes the possibility of providing such access when required by law or believed to be necessary or appropriate to comply with applicable laws and lawful requests and legal process (Privacy Policy, 2018). In principle, this could be seen at odds with the rationale behind blockchain technology, which restricts access to data for those who are not part of the network.

### Education and Awareness of the Potential Risks

Individuals should be encouraged to carefully weigh the benefits and risks of getting engaged in a DNA data marketplace. Moreover, raising awareness regarding the implications and possible consequences of personal genomic data sharing for the individuals and their family members is essential (Shabani and Borry, 2015). Currently, the potential concerns regarding genomic data sharing in the conventional research settings are being investigated (Middleton et al., 2018). However, the similar studies and educational materials in the context of data sharing in DNA data marketplace are missing.

Previously, in the context of Personal Genome Project (PGP), following educational videos have been required for those who agreed to share their genome publicly. In addition, the requirements such as higher level of education has been expected from volunteers of PGP (Reuter et al., 2018). Although this can be seen as one way to mitigate the concerns regarding awareness about the associated risks with such data sharing, but it may lead to biasing the sample of participants and work against diversity.

Moreover, the associated risks with sharing data through DNA data marketplace are not fully known yet. It is expected that some of the concerns such as those related to risks for privacy emerge only in the future and due to technological advances. The participants therefore should be aware of unknown risks.

## Concluding Remarks

The emerging DNA Data marketplaces are promising to introduce a fair model of data sharing among individuals and the interested parties such as pharmaceutical and biotechnology companies. They encourage the individuals to directly take part in sharing their data and practice their ownership rights regarding their DNA information. However, our analysis showed that developing DNA data marketplace raises concerns about consent and privacy and may have externalities for public-funded research that do not offer incentives.

One of the main arguments of developing DNA data marketplace is to empower individuals to directly share their data and control who can have access to data. In essence, empowerment of the individuals by enabling them to actively be involved in management of their personal health information has recently received an increasing attention. For example, The European Data Protection Supervisor published in October 2016 an opinion on this subject and recognized the potential of Personal Information Management Systems (PIMS) as one approach for effectively implementing citizens’ rights on their personal data at the practical level. PIMS “allow individuals to manage their personal data in secure, local or online storage systems and share them when and with whom they choose.” (European Data Protection Supervisor, 2016).

DNA data marketplace could be seen as an example of such approach, aiming for involving individuals in managing how to share their health data and with whom. However, in order to truly empower patients and individuals, it is crucial to ensure that they are adequately informed about the limitations on controlling their data once have been shared and accessed by companies and interested parties. In addition, the companies should develop fair and transparent policies on issues related to consent withdrawal in the view of offering tokens, shares, etc. in exchange for data.

Moreover, in discussions related to DNA marketplace, the attentions should be paid to the fact that human beings are relational beings sharing a lot of genetic details with others, and in particular family members. In particular, since genetic data carry family connections, the implications of data donation and receiving financial incentives for family members should be taken into considerations. Currently, the discussions related to consent and withdrawal mainly limited to the concerns related to individual rights in such data donation, and do not sufficiently address the pertinent interests of the family members. On practical level, it is also crucial to investigate how far family members should/can be involved in the process of personal genomic data sharing, including giving informed consent. Notably, in the context of genetic data, the applicable legal frameworks for personal data protection are predominantly limited to recognizing individuals as “data subjects” and do not extend to the family members.

In addition, in promoting the notions of self-interest and individual empowerment, values such as altruism and solidarity in the society should not be undermined (Prainsack, 2018). This is particularly may appear concerning to traditional biomedical research which relies on altruistic participation of the individuals to advance research as a public good. Moreover, offering monetary incentives may be considered as commodifying human resources, which has been extensively debated to date, as it may lead to undermining individuals’ dignity.

Notably, the success of data collection through such marketplaces is hinged on attracting a large number of participants; otherwise it would be hard to foresee a significant impact on the current way the medical research has been performed. It should be noted that currently some of other non-profit data sharing platforms such as DNA.Land that enables individuals to share their own genome- succeeded in collecting more than 150,000 genomes (Check Hayden, 2015). Therefore, the scalability of DNA data marketplaces may be seen as an achievable goal. Moreover, developing DNA Data Marketplaces and recruiting individuals directly may be considered as a solution to the problem of lack of diversity among study groups in biomedical sciences. The future studies are needed to survey the participants in such marketplaces and examine the level of diversity in terms of nationality, ethnicity, gender, and the like.

Finally, the use of the terms such as data ownership, buying and selling data, and data control in the context of personal genomic and health data should be thoroughly scrutinized, as such claims are surrounded by legal and practical uncertainties (Blasimme et al., 2018). One pertinent question is how the monetary value of DNA data can be estimated, and how this can be ethically and legally enforced (McNulty, 2018). EncrypGen declared that the price of access to data would be decided by the open market, while LunaDNA proposes different pricing for non-profits and corporations. In a recently published paper, LunaDNA presented a new model for research in which participants are issued US Securities and Exchange Commission (SEC)-qualified shares in whatever database holds their data. Thereby, “as shareholders, the participants would be eligible to receive commercial proceeds generated by mining their datasets, effectively transforming them from research subjects to partners.”(Curtis and Hereward, 2018; Kain et al., 2019).

This calls attention to the necessity of developing adequate guidelines, policies (soft-governance tools), and regulations in order to ensure both ethical and legal underpinnings of DNA data marketplaces as well as transparency and fairness of the procedure. That said, the existing national and European regulations regarding personal data protection and consumer protection provide general framework for some aspects of data collection and processing by such data marketplaces, including in relation with consent, data portability, and transparency of data processing.

## Author Contributions

EA and MS both contributed to the structuring, drafting, and revising the manuscript.

## Conflict of Interest

The authors declare that the research was conducted in the absence of any commercial or financial relationships that could be construed as a potential conflict of interest.
